# Indications for haematopoietic cell transplantation for haematological diseases, solid tumours and immune disorders: current practice in Europe, 2022

**DOI:** 10.1038/s41409-022-01691-w

**Published:** 2022-05-19

**Authors:** John A. Snowden, Isabel Sánchez-Ortega, Selim Corbacioglu, Grzegorz W. Basak, Christian Chabannon, Rafael de la Camara, Harry Dolstra, Rafael F. Duarte, Bertram Glass, Raffaella Greco, Arjan C. Lankester, Mohamad Mohty, Bénédicte Neven, Régis Peffault de Latour, Paolo Pedrazzoli, Zinaida Peric, Ibrahim Yakoub-Agha, Anna Sureda, Nicolaus Kröger, for the European Society for Blood and Marrow Transplantation (EBMT)

**Affiliations:** 1 Department of Haematology, Sheffield Teaching Hospitals NHS Foundation Trust, Sheffield, UK; 2 EBMT Executive Office, Barcelona, Spain; 3 University of Regensburg, Regensburg, Germany; 4 Department of Hematology, Transplantation and Internal Medicine, University Clinical Centre, Medical University of Warsaw, Warsaw, Poland; 5 Institut Paoli Calmettes Comprehensive Cancer Center & Centre d’Investigations Cliniques en Biothérapies Inserm CBT-1409, Marseille, France; 6 Hematology, Hospital de la Princesa, Madrid, Spain; 7 Laboratory of Hematology, Department of Laboratory Medicine, Radboud University Medical Center, Nijmegen, The Netherlands; 8 Hospital Universitario Puerta de Hierro Majadahonda—Universidad Autónoma de Madrid, Madrid, Spain; 9 Klinik für Hämatologie und Stammzelltransplantation, HELIOS Klinikum Berlin-Buch, Berlin, Germany; 10 Haematology and Bone Marrow Transplant Unit, IRCCS San Raffaele Scientific Institute, Milano, Italy; 11 Willem-Alexander Children’s Hospital, Leiden University Medical Center, Leiden, The Netherlands; 12 Sorbonne University, Saint-Antoine Hospital (AP-HP), INSERM UMRs 938, Paris, France; 13 Pediatric Immuno-hematology Unit, Necker Children Hospital, Assistance Publique Hopitaux de Paris, Université de Paris Cité, Paris, France; 14 Saint-Louis Hospital, Paris Diderot University, Paris, France; 15 Fondazione IRCCS Policlinico San Matteo and University of Pavia, Pavia, Italy; 16 Department of Hematology, University Hospital Centre Zagreb and School of Medicine, University of Zagreb, Zagreb, Croatia; 17 CHU de Lille, Univ Lille, INSERM U1286, Infinite, 59000 Lille, France; 18 Institut Català d’Oncologia—Hospital Duran i Reynals, Barcelona, Spain; 19 University Medical Center Hamburg, Hamburg, Germany

**Keywords:** Haematological cancer, Haematopoietic stem cells

## Abstract

For over two decades, the EBMT has updated recommendations on indications for haematopoietic cell transplantation (HCT) practice based on clinical and scientific developments in the field. This is the eighth special EBMT report on the indications for HCT for haematological diseases, solid tumours and immune disorders. Our aim is to provide general guidance on HCT indications according to prevailing clinical practice in EBMT countries and centres. In order to inform patient decisions, these recommendations must be considered in conjunction with the risk of the disease, risk of HCT procedure and non-transplant strategies, including evolving cellular therapies. HCT techniques are constantly evolving and we make no specific recommendations, but encourage harmonisation of practice, where possible, to ensure experience across indications can be meaningfully aggregated via registry outputs. We also recommend working according to JACIE accreditation standards to maintain quality in clinical and laboratory components of practice, including benchmarking of survival outcomes. Since the last edition, the COVID-19 pandemic has affected clinical decision making and activity across indications. Although the full impact of the pandemic is yet to be determined, we recommend that decision making across indications is delivered with ongoing reference to EBMT and national COVID-19 guidance, in accordance with current local conditions.

## Introduction

This is the eighth report from the European Society for Blood and Marrow Transplantation (EBMT) covering indications for haematopoietic cell transplantation (HCT) according to prevailing clinical practice in EBMT countries and centres [1]. For over two decades, EBMT has considered changes in HCT practice alongside developments in non-transplant treatments. As in previous editions, these 2022 recommendations are based upon clinical trials, registry data, and the opinion of EBMT experts from the board, scientific council and relevant working parties, but not upon a formal extensive or systematic review of the literature. They aim to provide general guidance on transplant indications to inform individual patient decisions by the multidisciplinary team (MDT). They must be considered in conjunction with the risk of the disease status, the likelihood of the successful outcome of HCT, assessment of patient co-morbidities and estimation of treatment-related mortality (TRM) risk alongside the results of non-transplant strategies. Besides potential survival benefits, assessment must include quality of life and late effects. The recommendations are not intended to be used to choose a particular transplant protocol, conditioning regimen or stem cell source, but we encourage harmonisation of practice, where possible, to ensure meaningfully aggregated experience across indications via registry outputs.

Since the last update, we have experienced the Coronavirus disease-19 (COVID-19) pandemic, which has affected HCT activity for a variety of reasons, from risks to patients, availability of donors and stem cell products, through to maintenance of staffing and services [2]. Waves of the pandemic impacted variably across our geographical regions, requiring broader public health measures, including the uptake of vaccination. From an early stage, there was recognition that outcomes following HCT and CAR-T cell therapies were poorer in patients in whom SARS-2-CoV infection was detected and also a response to vaccination is reduced and variable in the HCT setting. The EBMT regularly updates recommendations for SARS-COv-2 management and vaccination (https://www.ebmt.org/covid-19-and-bmt). Consequently, there has been a temporary reduction in HCT rates in some indications, with necessary prioritisation between indications and delays in treatment that may have impacted upon outcomes of HCT. Although the full impact of the pandemic is yet to be determined and our understanding and evidence-base is evolving, we recommend that decision making across indications is made by an MDT with reference to EBMT and other national guidance in relation to COVID-19, and in accordance with current local conditions.

This guidance does not primarily intend to cover cellular therapies, but makes reference to the application of CAR-T cells as a treatment option alongside HCT in acute lymphoblastic leukaemia (ALL) and lymphomas, and, more developmentally, in multiple myeloma, chronic lymphocytic leukaemia and acute myeloid leukaemia.

Importantly, we recognise that there is an overlap between adult and paediatric indications, particularly in the ‘teenager and young adult’ (TYA) group, and definitions of paediatric and TYA (and therefore ‘adult’ care) vary internationally. Despite the age cut-offs, which are also influenced by EBMT registry definitions, the indications should be interpreted with flexibility, particularly in the TYA age group and some ‘paediatric’ and TYA indications may occasionally extend into older adult age groups. Given this consideration, we have combined ‘Inherited diseases’ into considerations for all ages, although the predominant use of HCT will be for the paediatric age group.

## Transplant categorisation, definitions and factors

### Haematopoietic cell transplant

HCT refers to any procedure where haematopoietic stem cells (HSC) of any donor type and source are given to a recipient with the intention of repopulating and replacing the haematopoietic system in total or in part. HSC for HCT can be derived from bone marrow (BM), peripheral blood (PB) or cord blood (CB).

### Donor categories and stem cell sources

Donor type is categorised as autologous, syngeneic and allogeneic, the latter being either related or unrelated. Beyond HLA-matched related (i.e., sibling) donors (MSD), a well-matched unrelated donor (MUD) is defined as a 10/10 identical unrelated donor (UD) based on HLA high-resolution typing for class I (HLA-A, -B, -C) and II (HLA-DRB1, -DQB1). A mismatched unrelated donor (MMUD) refers to a UD mismatched in at least one antigen or allele at HLA-A, -B, -C, -DR or -DQ and a haploidentical donor refers to one haplotype mismatched donor, most frequently being a family donor. Criteria for UD selection have been proposed [3, 4], but are beyond the scope of these recommendations and incorporation into clinical practice will depend on the effort from donor registries and transplant centres balanced against strategies to incorporate mismatched alternative donors (MMAD), including haploidentical donors, into practice.

Recent developments include non–T-cell-depleted haploidentical HCT, mainly with post-transplant cyclophosphamide (PTCy) as graft-versus-host disease (GVHD) prophylaxis that has led to a reduction in chronic GVHD and TRM [5] in this setting. In parallel, results of unrelated and mismatched unrelated transplants are continually improving, and studies (mostly registry based) have shown similar outcomes of 10/10 HLA compatible unrelated transplants compared with HCT from an MSD [2]. Moreover, several retrospective studies have shown similar results for haploidentical HCT with PTCy to MSD and MUD [6–10]. However, additional studies are needed to confirm these findings for particular indications. Therefore, in this current version, we continue to combine the recommendations for MMAD, incorporating CB, haploidentical and MMUD into a single category separate from well-matched related and UD. Beyond this general approach, the relative value of the various modalities is described and addressed in more detail below in sections for the relevant indications.

Transplant decisions derive not only from sound clinical and scientific evidence but also from each centre’s research priorities, local expertise, cost considerations and ease of access to particular transplant modalities, along with individual patient and donor preference. Ultimately, we recommend all aspects are brought together and documented in an MDT meeting.

## Categorisation of type of indication for transplant procedures

EBMT indications are classified into four categories (Table 1), to describe the levels of evidence and recommendations for types of transplants and different indications.

**Table 1:** EBMT categorisation of type of indication for transplant procedures and strength of evidence.

Categories	Settings where HCT ought to be performed
Standard of care (S)	Indications reasonably well defined and results compare favourably (or are superior) to those of non-transplant treatment approaches. Obviously, defining an indication as the standard of care does not mean an HCT is necessarily the optimal therapy for a given patient in all clinical circumstances. ‘Standard of care’ transplants may be performed in a specialist centre with experience in HCT and an appropriate infrastructure as defined by the JACIE standards.
Clinical option (CO)	Indications for which the results of small patient cohorts show efficacy and acceptable toxicity of the HCT procedure, but confirmatory randomised studies are missing, often as a result of low patient numbers. The broad range of available transplant techniques combined with the variation of patient factors such as age and co-morbidity makes interpretation of these data difficult. Our current interpretation of existing data for indications placed in this category supports that HCT is a valuable option for individual patients after careful discussions of risks and benefits with the patient, but that for groups of patients the value of HCT needs further evaluation. Transplants for indications under this heading should be performed in a specialist centre with major experience in HCT with an appropriate infrastructure as defined by JACIE standards.
Developmental (D)	Indications when the experience is limited, and additional research is needed to define the role of HCT. These transplants should be done within the framework of a clinical protocol, normally undertaken by transplant units with acknowledged expertise in the management of that particular disease or that type of HCT. Protocols for D transplants will have been approved by local research ethics committees and must comply with current international standards. Rare indications where formal clinical trials are not possible should be performed within the framework of a structured registry analysis, ideally an EBMT non-interventional/observational study. Centres performing transplants under this category should meet JACIE standards.
Generally not recommended (GNR)	Comprises a variety of clinical scenarios in which the use of HCT cannot be recommended to provide a clinical benefit to the patient, including early disease stages when results of conventional treatment do not normally justify the additional risk of an HCT, very advanced forms of a disease in which the chance of success is so small that does not justify the risks for patient and donor, and indications in which the transplant modality may not be adequate for the characteristics of the disease. A categorisation as GNR does not exclude that centres with particular expertise on a certain disease can investigate HCT in these situations. Therefore, there is some overlap between GNR and D categories, and further research might be warranted within prospective clinical studies for some of these indications.
Grade	*Strength of the evidence supporting the assignment of a particular category*
Grade I	Evidence from at least one well-executed randomised trial.
Grade II	Evidence from at least one well-designed clinical trial without randomisation; cohort or case-controlled analytic studies (preferably from more than one centre); multiple time-series studies; or dramatic results from uncontrolled experiments.
Grade III	Evidence from opinions of respected authorities based on clinical experience, descriptive studies, or reports from expert committees.

### Transplant indications in adults

The updated 2022 classification of HCT procedures in adults is shown in Table 2.

**Table 2:** Proposed classification of transplant indications for adults—2022.

Disease	Disease status	MSD allo	MUD allo	MMAD allo	Auto	CAR-T
*Haematological malignancies*						
AML^a^	CR1 (favourable risk and MRD–)^b^	GNR/II	GNR/II	GNR/II	CO/I	
	CR1 (favourable risk and MRD+)^b^	S/II	CO/II	CO/II	GNR/II	
	CR1 (intermediate risk)^b^	S/II	CO/II	CO/II	CO/I	
	CR1 (adverse risk)^b^	S/II	S/II	S/II	GNR/I	
	CR2	S/II	S/II	S/II	CO/II	
	APL Molecular CR2	S/II	CO/II	GNR/III	S/II	
	Relapse or refractory	CO/II	CO/II	CO/II	GNR/III	
ALL^a^	Ph (–), CR1 (standard risk and MRD–)^b^	GNR/II	GNR/II	GNR/III	CO/III	
	Ph (–), CR1 (standard risk and MRD+)^b^	S/II	CO/II	CO/II	GNR/II	CO/II
	Ph (–), CR1 (high risk)^b^	S/II	S/II	CO/II	GNR/III	
	Ph (+), CR1 (MRD–)	S/II	S/II	CO/II	CO/III	
	Ph (+), CR1 (MRD+)	S/II	S/II	S/II	GNR/II	
	CR2	S/II	S/II	S/II	GNR/II	
	Relapse or refractory	CO/II	CO/II	CO/II	GNR/III	
CML	1st CP, failing 2nd or 3rd line TKI	S/II	S/II	CO/III	GNR/II	
	Accelerated phase, blast crisis or >1st CP	S/II	S/II	CO/II	GNR/III	
Myelofibrosis	Primary or secondary with an intermediate-2 or high DIPSS score	S/II	S/II	S/III	GNR/III	
MDS	Very low and low-risk (IPSS-R)	CO/II	CO/II	CO/II	GNR/III	
	Intermediate-risk without additional factors^c^ (IPSS-R)	CO/II	CO/II	CO/II	CO/II	
	Intermediate-risk with additional factors^c^ (IPSS-R)	S/II	S/II	S/II	GNR/III	
	High-, very high-risk (IPSS-R)	S/II	S/II	S/II		
	sAML in CR1 or CR2	S/II	S/II			
CMML	CMML-2 or MP-CMML	S/II	S/II	S/II	GNR/III	
	CMML-0 or CMML-1 with additional risk factors^d^	S/II	S/II	S/II	GNR/III	
CLL	Poor risk disease refractory or relapsing after at one line of prior therapy (Richter’s transformation excluded)	CO/II	CO/II	GNR/III	GNR/III	CO/II
	Richter transformation	S/II	S/II	S/II	GNR/III	CO/II
LBCL	CR1 (intermediate/high IPI at diagnosis)	GNR/III	GNR/III	GNR/III	CO/I	GNR/III
	Untested relapse	GNR	GNR	GNR	GNR	S/I
	Chemosensitive early relapse, ≥CR2	CO/II	CO/II	D/III	CO/I	S/II
	Chemosensitive late relapse, ≥CR2	CO/II	CO/II	D/III	S/II	CO/II
	Chemosensitive relapse after auto-HSCT failure	CO/II	CO/II	CO/III	GNR/III	S/II
	Refractory disease	CO/II	CO/II	CO/III	GNR/I	S/I
	Primary CNS lymphoma	GNR/III	GNR/III	GNR/III	S/II	D/III
FL	CR1, untransformed	GNR/III	GNR/III	GNR/III	GNR/II	GNR/III
	CR1, transformed into high-grade lymphoma	GNR/III	GNR/III	GNR/III	CO/III	GNR/II
	Chemosensitive relapse, ≥CR2	CO/III	CO/III	GNR/III	S/II	GNR/III
	≥CR2 after auto-HSCT failure	S/II	S/II	D/III	GNR/III	CO/II
	Refractory	CO/II	CO/II	CO/III	GNR/III	CO/II
MCL	CR1	GNR/III	GNR/III	GNR/III	S/I	GNR/III
	CR/PR >1, no prior auto-HCT	CO/III	CO/III	D/III	CO/II	S/II
	CR/PR >1, after prior auto-HCT	CO/II	CO/II	CO/III	GNR/II	S/II
	Refractory	CO/II	CO/II	CO/III	GNR/II	S/II
WM	CR1	GNR/III	GNR/III	GNR/III	GNR/III	GNR/III
	Chemosensitive relapse, ≥CR2	GNR/III	GNR/III	GNR/III	CO/II	GNR/III
	Poor risk disease	CO/II	CO/II	D/III	GNR/III	GNR/III
PTCL	CR1	CO/II	CO/II	GNR/III	CO/II	GNR/III
	Chemosensitive relapse, ≥CR2	S/II	S/II	CO/III	CO/II	GNR/III
	Refractory	CO/II	CO/II	CO/III	GNR/II	GNR/III
Primary CTCL	EORTC/ISCL Stages I–IIA (early)	GNR/III	GNR/III	GNR/III	GNR/III	GNR/III
	EORTC/ISCL Stages IIB–IV (advanced)	CO/III	CO/III	D/III	GNR/III	GNR/III
HL	CR1	GNR/III	GNR/III	GNR/III	GNR/I	GNR/III
	Chemosensitive relapse, no prior auto-HCT	D/III	D/III	GNR/III	S/I	GNR/III
	Chemosensitive relapse, after prior auto-HCT	S//II	S/II	S/II	CO/III	GNR/III
	Refractory	D/II	D/II	D/III	CO/III	GNR/III
MM	Upfront standard risk	CO/II	CO/II	GNR/III	S/I	
	Upfront high risk	S/III	S/III	CO/II	S/I	
	Chemosensitive relapse, prior auto-HCT	CO/II	CO/II	CO/II	S/II	GNR/III
	Refractory/relapse after three lines of prior therapy including an immunomodulatory agent, a proteasome inhibitor and an anti-CD38					S/II
AL		CO/III	CO/III	GNR/III	CO/II	
*Other diseases*						
Acquired SAA and AA/PNH	Newly diagnosed	S/II	CO/II	GNR/III	NA	
	Relapsed/refractory	S/II	S/II	CO/II	NA	
Haemolytic PNH		GNR/II	GNR/II	GNR/II	NA	
Constitutional BMF syndromes/SAA^e^		S/II	S/II	CO/II	NA	
Breast Ca	Adjuvant high risk, selected population	NA	NA	NA	D/CO/I	
	Metastatic, chemosensitive	D/II	NA	NA	D/CO/II	
Germ cell tumours	Second line, high risk	GNR/III	NA	NA	CO/II	
	Primary refractory, second and further relapse	GNR/III	NA	NA	S/II	
Ovarian Ca	High risk/recurrent	GNR/II	NA	NA	GNR/I	
Medulloblastoma	Post-surgery, high risk/recurrent disease	NA	NA	NA	CO/III	
Small cell lung Ca	Limited	NA	NA	NA	GNR/I	
Soft tissue Sa	Advanced	D/III	NA	NA	D/II	
Ewing’s Sa	Locally advanced/metastatic, chemosensitive	D/III	NA	NA	CO/II	
Renal cell Ca	Metastatic, refractory to conventional treatments	D/II	NA	NA	NA	
Colorectal Ca, pancreatic Ca, other selected solid tumours	Metastatic, refractory to conventional treatments	D/III	NA	NA	NA	
Multiple sclerosis	Highly active RR-MS failing DMT	D/III	GNR/III	GNR/III	S/I	
	Progressive MS with AIC, and Aggressive MS^f^	D/III	GNR/III	GNR/III	CO/II	
	Progressive MS without AIC	GNR/III	GNR/III	GNR/III	GNR/III	
Systemic sclerosis		D/III	GNR/III	GNR/III	S/I	
SLE		D/III	GNR/III	GNR/III	CO/II	
Crohn’s disease		D/III	D/III	D/III	CO/II	
Rheumatoid arthritis		D/III	GNR/III	GNR/III	CO/II	
JIA		CO/II	CO/II	CO/III	CO/II	
Monogenic AD		CO/II	CO/II	CO/III	GNR/II	
Vasculitis	ANCA+ve, BD, Takayasu, others	GNR/III	GNR/III	GNR/III	CO/II	
PM-DM		GNR/III	GNR/III	GNR/III	CO/II	
Autoimmune cytopenias		CO/II	CO/II	CO/III	CO/II	
Neuromyelitis optica		D/III	D/III	D/III	CO/II	
CIDP, MG and SPS		GNR/III	GNR/III	GNR/III	CO/II	
Type 1 diabetes		GNR/III	GNR/III	GNR/III	D/II	
RCD type II		GNR/III	GNR/III	GNR/III	CO/II	
Primary ID		CO/II	CO/II	CO/II	NA	

This classification does not cover patients for whom a syngeneic donor is available.

*AA* aplastic anaemia, *AD* autoimmune disorders, *AIC* active inflammatory component, *AL* amyloidosis, *ALL* acute lymphoblastic leukaemia, *Allo* allogeneic transplantation, *AML* acute myeloid leukaemia, *APL* acute promyelocytic leukaemia, *Auto* autologous transplantation, *Ca* cancer or carcinoma, *CAR-T* chimeric antigen receptor T cells, *CIDP* chronic inflammatory demyelinating polyneuropathy, *CLL* chronic lymphocytic leukaemia, *CML* chronic myelogenous leukaemia, *CMML* chronic myelomonocytic leukaemia, *CO* clinical option (can be carried after careful assessment of risks and benefits), *CP* chronic phase, *CR1, 2, 3* first, second, third complete remission, *CTCL* cutaneous T-cell lymphoma, *D* developmental (further trials are needed), *DIPSS* dynamic international prognostic score system, *DMT* disease-modifying treatments, *FL* follicular lymphoma, *GNR* generally not recommended, *HL* Hodgkin lymphoma, *HCT* haematopoietic cell transplantation, *ID* immunodeficiency, *IPI* international prognostic index, *IPSS-R* revised International Scoring System, *JIA* juvenile idiopathic arthritis, *LBCL* large B-cell lymphoma, *MCL* mantle cell lymphoma, *MDS* myelodysplastic syndromes, *MG* myasthenia gravis, *MM* multiple myeloma, *MMAD* mismatched alternative donors (cord blood, haploidentical and mismatched unrelated donors), *MP-CMML* myeloproliferative CMML, *MRD* minimal residual disease, *MS* multiple sclerosis, *MSD* matched sibling donor, *MUD* well-matched unrelated donor (8/8, 10/10, or 9/10 if mismatched is in DQB1), *NA* not applicable, *PM-DM* polymyositis-dermatomyositis, *PNH* paroxysmal nocturnal haemoglobinuria, *PR* partial remission, *RA* refractory anaemia, *RAEB* refractory anaemia with excess blasts, *RCD* refractory coeliac disease, *RCMD* refractory cytopenia with multilineage dysplasia, *RR-MS* relapsing-remitting multiple sclerosis, *S* standard of care (generally indicated in suitable patients), *Sa* sarcoma, *SAA* severe aplastic anaemia, *sAML* secondary acute myeloid leukaemia, *SLE* systemic lupus erythematosus, *SPS* stiff person syndrome, *TCL* T-cell lymphoma, *TKI* tyrosine kinase inhibitors, *WM* Waldenström macroglobulinemia.

^a^Some centres consider older age (e.g., >60 years) as a criterion for high-risk disease in decision making for allogeneic HSCT for AML or ALL. Beyond transplant indications, maintenance therapy after transplant is being increasingly used with the aim of improving survival outcomes (e.g., FLT3 inhibitors in FLT3-ITD AML [346]).

^b^Categories are based on number of white blood cells, cytogenetics and molecular markers at diagnosis and time to achieve remission (see text).

^c^Additional factors include >5% marrow blasts, poor karyotype, profound cytopenias (i.e., Hb <80 g/L, ANC <0.8 × 10^9^/L, platelets <50 × 10^9^/L), or severe BM fibrosis.

^d^Additional high-risk gene mutations (ASXL1, RUNX1, SETBP1, N-RAS), severe cytopenia or transfusion dependency, excessive proliferative features or extramedullary involvement.

^e^Constitutional SAA includes Fanconi anaemia, dyskeratosis congenita, Blackfan–Diamond anaemia and other inborn bone marrow failure syndromes (see also the section and table for paediatric indications).

^f^Aggressive MS as per Menon et al. [347].

#### Acute myeloid leukaemia (AML)

As per EBMT’s last activity survey, AML accounts for more than one-third of allo-HCT transplants [2]. The heterogeneity of AML, response to treatment and associated morbidity and TRM of HCT mandates risk stratification based on patient, disease, and transplant parameters [11, 12], which has led to significant improvement in outcomes for AML in recent years [13]. Genetic risk factors form the basis of modern prognostication for predicting outcome in AML patients and guide decisions in relation to HCT as a post-remission treatment modality [14–16], including cytogenetics [14, 15, 17], molecular mutations [16, 18, 19] and post-induction measurable residual disease (MRD) status [20–22]. Alongside hematopoietic cell transplantation-specific co-morbidity index (HCT-CI) [23], data-mining-based machine-learning algorithms may improve prediction of day +100 mortality post-HCT [24]. Transplant techniques, including improvements in supportive care, prevention and treatment of infective complications and reduced intensity conditioning, have led over the last 20 years to almost a 50% reduction in TRM [14] and increasing treatment of patients over the age of 65 years [25].

Overall, allo-HCT in first complete remission (CR1) is recommended for adverse and intermediate-risk AML as defined by the European LeukemiaNet (ELN) risk stratification that is based on cytogenetics and mutational phenotype, while HCT is not recommended for AML patients with favourable disease [1, 14]. Specifically, HCT in CR1 is recommended for AML patients with adverse cytogenetics [1, 14] and patients with normal cytogenetics with unmutated NPM1 plus high FLT3-ITD allele ratio or those harbouring RUNX1, ASXL1, or TP53 mutations. However, HCT is not recommended in CR1 in the favourable risk group, including AML patients with core-binding factor (CBF) that are associated with translocations *t*(8;21), inv(16) or t(16;16) and NPM1 mutation plus wild-type FLT3-ITD or FLT3 with low-allele ratio (<0.5) (although this may be controversial) [1, 14].

Allo-HCT may be effective in a subset of patients with primary refractory AML (defined as failure to achieve CR after two cycles of induction chemotherapy) and relapsing AML [26], especially those achieving CR2 [27]. However, patients with resistant disease should be considered for novel transplant approaches, including trials of CAR-T cells [19]. All types of donors should be considered for suitably fit, adverse risk patients with AML, while those with intermediate risk are usually transplanted using MSD or 10/10 UD [1].

AML patients in the favourable risk category may be considered for auto-HCT especially if they are MRD negative post induction therapy [1, 14], while patients with persistent MRD should be considered for allo-HCT [28]. Low levels of CBF fusion gene transcripts may persist after the end of treatment without affecting survival. However, failure to achieve a 3-log reduction in CBF fusion transcript after two cycles of chemotherapy is associated with a high risk of relapse and these patients might benefit from allo-HCT [29, 30]. Younger adults with NPM1-mutated AML and MRD positivity by RQ-PCR in the PB after two cycles of chemotherapy have higher rates of relapse and should be considered for allo-HCT [31]. Standard-risk younger patients with NPM1-unmutated AML with positive MRD by multi-parametric flow cytometry seem to benefit from HCT in CR1 [32]. Although controversial, auto-HCT [1] may also be considered for intermediate-risk patients and offered for AML-M3 patients achieving CR2 and MRD negativity [1].

#### Acute lymphoblastic leukaemia (ALL)

As per EBMT’s latest activity survey, ALL is the second most common indication for allo-HCT, accounting for 17% of cases [2], with Ph-negative ALL in CR as the major proportion [33]. Having been originally established on donor versus no donor studies and subsequent meta-analyses, allo-HCT continues to be the standard of care in high-risk ALL patients defined by conventional risk factors and MRD status (i.e., adverse cytogenetics, slow remitters, after failure or inability to receive a paediatric-inspired regimen) and in relapsed ALL, but is not indicated for standard-risk ALL, especially if MRD negative [34–38].

Results of allo-HCT from UD are constantly improving as those from non–T-cell-depleted haploidentical donors, as alternatives for transplant-eligible ALL patients lacking a MSD [39, 40]. Importantly, in recent years, the treatment paradigm for primary refractory and relapsed Philadelphia (Ph) negative ALL has been revolutionised with the introduction of blinatumomab, a first-in-class bispecific T-cell engager anti-CD19 monoclonal antibody (mAb) and the drug conjugate, inotuzumab ozogamicin, an anti-CD22-calicheamicin mAb. In addition, anti-CD19 CAR-T cell therapy was recently approved for patients up to 25 years old [41–43]. This therapeutic progress is additional to Ph-positive ALL by tyrosine kinase inhibitors (TKIs) [44]. Furthermore, MRD monitored throughout the therapy pathway, guides treatment and is important for decision making [45]. Whether standard-risk ALL patients achieving MRD negativity in CR1 on paediatric-inspired treatment protocols should undergo HCT is a matter of debate, while those with persistent MRD post-consolidation should proceed to allo-HCT [46–49].

High-risk cytogenetics at diagnosis is an indication for allo-HCT, even in MRD-negative patients, at least until additional studies are available [50–52]. Adverse cytogenetics include, but, are not limited to, low hypodiploidy, KMT2A (previously MLL) translocations, *t*(8;14), complex karyotype (≥5 chromosomal abnormalities) and Ph-positive ALL.

Allo-HCT is also indicated for adult ALL patients in CR2 or beyond [53] and may rescue a subset of patients with resistant disease responding to novel agents such as blinatumomab, inotuzumab ozogamicin and/or CAR-T cells [54].

Anti-CD19 CAR-T cell therapy is approved for patients up to 25 years of age based on the ELIANA study [55], and more recently for adult ALL based on the ZUMA-3 trial [56]. Most trials of CAR-T cells in relapsed/refractory ALL demonstrate impressive response rates, with >70% of patients achieving CR regardless of cytogenetic background, prior therapies, or age [57]. Prognostic factors associated with higher remission rates and better outcome in adult ALL include lower disease burden (assessed by BM blast count), lower LDH and higher platelet count prior to lymphodepletion [58, 59]. TP53 mutations are associated with worse outcome [59]. Importantly, conditioning with fludarabine and cyclophosphamide is superior to cyclophosphamide alone [58]. Due to the time delay between detection of relapse and infusion of CAR-T cells, bridging therapy is often necessary, although results may be inferior in patients previously treated with blinatumomab [60] and toxicity may be higher in patients with a previous allo-HCT [59].

Relapse after CAR-T cell therapy occurs in 30–50% of patients (with CD19 negative relapse in up to 40%) [57, 61]. The question of post-CAR-T cell consolidation with allo-HCT is still open and data are controversial. Many centres undertake allo-HCT for adult ALL patients following CAR-T cell treatment even in an MRD-negative remission. In any event, patients with molecular MRD positivity following CAR-T cell therapy, loss of B-cell aplasia and without a previous HCT are candidates for consolidative allo-HCT post CAR-T cell infusion [61].

For Ph-positive ALL, allo-HCT is still indicated in CR1 [62]. Post-transplant TKIs have shown to reduce relapse rates and should therefore be considered [62]. Auto-HCT is an option for patients achieving MRD negativity [63]. However, emerging data show that patients achieving deep MRD negativity adequately treated with TKIs in combination with mAbs may avoid HCT [64, 65]. Allo-HCT is indicated for Ph-like ALL, due to the described poor outcome irrespective of MRD status [66].

In contrast to B-ALL, salvage options in T-ALL are limited and consolidation with HCT in CR1 should be considered in high-risk patients. Similarly to B-ALL, MRD is a key predictor of relapse [7, 67]. Other high-risk features include early T-precursor immunophenotype [68] and adverse cytogenetics [69, 70]. Complex cytogenetics is associated with poor outcome, while NOTCH1 and/or FBXW7 mutations are associated with improved outcomes [67, 70].

With respect to auto-HCT, in the donor versus no donor studies and subsequent meta-analyses, no beneficial effect was observed [35–37]. The question as to whether auto-HCT should be revisited for MRD-negative patients has been discussed [63].

#### Chronic myeloid leukaemia (CML)

Since the advent of TKIs, allo-HCT is not recommended as a first-line treatment in CML. In the vast majority of CML patients in chronic phase (CP), imatinib or second-generation TKIs, such as dasatinib, nilotinib or bosutinib, should be the first-line therapy. Some patients achieving molecular remission with TKI treatment have remained in molecular remission for long periods after cessation of the drug and complete discontinuation of TKI could be obtained in about 40%, but it remains to be seen in the long term whether these patients are cured [71, 72].

Patients who fail first-line therapy according to ELN guidelines should start on second-line TKI therapy. Donor search should start in patients who fail two lines of TKI. They should receive treatment with third-line TKI depending on ABL mutation analyses and are candidates to proceed to allo-HCT as soon as possible whilst in optimal response if EBMT risk score is 0–1 or, in the case of a prior loss of cytogenetic or haematological response to second-line TKI, if EBMT risk score is 0–4. If there is no haematological response to second-line treatment, patients are candidates for allo-HCT with any EBMT risk score. Patients with ABL mutations resistant to third-generation TKI or with T315I mutation are candidates to undergo HCT with any EBMT risk score after failing second or third-line TKI.

Patients in advanced phase CML referred for an HCT could receive TKI or TKI± intensive therapy as preparation for HCT, which should be performed as soon as possible after achieving CP2 without the need for deep cytogenetic or molecular responses.

Patients with a syngeneic donor are always candidates for HCT with standard conditioning. Auto-HCT is generally not recommended outside clinical trials.

Overall, the main goal of CML therapy should be to prevent progression to the advanced phase or >CP1, as outcomes post-transplantation are worse in those situations. Patients with CML should be followed closely and considered for HCT while still in CP1 if they fulfil the criteria described above.

#### Myeloproliferative neoplasms (MPNs)

Allo-HCT remains the only potential curative option for patients with MPNs. However, polycythaemia vera and essential thrombocythaemia are generally not indications for allo-HCT unless the disease has progressed to secondary myelofibrosis or secondary leukaemia [73–75]. Due to the lack of alternative therapeutic options, allo-HCT is a reasonable treatment for intermediate II and high-risk primary myelofibrosis (according to the Dynamic International Prognostic Index) [76]. In younger patients, transplantation is justified in individual cases classified as intermediate I, especially if unfavourable mutations such as EZH2 or ASXL1 or unfavourable cytogenetics are present [77, 78]. Experience of allo-HCT in patients with low-risk index disease is limited and remains controversial.

The introduction of JAK inhibitors in the treatment of primary myelofibrosis may help to improve constitutional symptoms and to reduce spleen size before HCT, but their definitive role needs to be determined [79]. Recent EBMT data suggest no disadvantage with pre-transplant exposure to ruxolitinib, and better outcomes after transplant in ruxolitinib responsive patients compared with patients with no response or loss of response to ruxolitinib [80]. A recent validated transplant risk score for myelofibrosis may be helpful in decision making [81].

Auto-HCT for MPN is generally not recommended outside clinical trials.

#### Myelodysplastic syndromes (MDS)

Allo-HCT is a curative option for patients with higher-risk MDS, but patient-related factors, especially age and co-morbidities, often impact upon feasibility of HCT. EBMT proposed a transplant-specific risk score for MDS patients [82], which, along with HCT-CI, may be used to judge the feasibility of allo-HCT and predict outcomes. In addition, disease characteristics that impact the risk of transformation into AML and survival need to be considered. The revised international prognosis scoring system (IPSS-R) is the most relevant system to assess disease prognosis [83]. This revised scoring system is based on marrow blast percentage, modified cytogenetic risk groups [84] and severity of cytopenias. It is an age-adjusted risk score [85] and classifies disease-related factors in five categories of risk; ‘very low’ (score ≤1.5), ‘low’ (>1.5≤3.0), ‘intermediate’ (>3≤4.5), ‘high’ (>4≤6), and ‘very high’ (>6). The time to 25% AML progression/median survival in the absence of therapy were reported as follows; not reached/8.8 years, 10.8/5.3 years, 3.2/3 years, 1.4/1.6 years and 0.7/0.8 years for patients in very low-, low-, intermediate-, high- and very high- risk categories, respectively. To simplify clinical use, patients can be categorised into three risk groups when considering allo-HCT: ‘lower-risk’ (LR-MDS) including low- and very-low-risk groups, ‘intermediate-risk’, and ‘higher-risk’ (HR-MDS) including high- and very-high-risk groups.

While allo-HCT is a standard of care in suitably fit patients with higher-risk MDS, decision making becomes more challenging for the intermediate IPSS-R group, where patients have a median survival of 3 years without treatment and 25% AML progression at 3.2 years. Patients in the intermediate IPSS-R risk group with >5% marrow blasts at diagnosis, poor karyotype, profound cytopenias (i.e., haemoglobin <80 g/L, neutrophil count <0.8 × 10^9^/L, platelet count <50 × 10^9^/L), or severe BM fibrosis should be considered for allo-HCT. Otherwise, patients in this risk group should be offered non-transplant options as first line of therapy [83, 86].

#### Chronic myelomonocytic leukaemia (CMML)

Allo-HCT represents the only potentially curative treatment option in CMML, but, due to reported high rates of TRM and post-transplant relapse, HCT is generally recommended for carefully selected patients with high-risk disease [83] and acceptable HCT-CI. However, younger patients stratified in low-risk categories may be referred for possible allo-HCT in presence of additional risk factors, such as gene mutations, severe cytopenia, transfusion dependency, excessive proliferative features or extramedullary involvement.

According to the 2016 WHO classification of myeloid neoplasms, CMML is classified in three stages: CMML-0 (blasts <5% in BM and <2% in PB, CMML-1 (blasts 5–9% in BM or 2–4% in PB) and CMML-2 (blasts 10–19% in BM or 5–19% in PB). There are two variants of the disease, ‘dysplastic’ and ‘proliferative’, depending on the circulating leukocyte count (≤13 × 10^9^/L and >13 × 10^9^/L, respectively) [87].

For the evaluation of disease-related factors, the use of a CMML-specific prognostic scoring system (CPSS) is recommended, possibly chosen among those including molecular information (CPSS-molecular, Groupe Francophone des Myelodysplasies, GFM, score, Molecular Mayo Score) [88].

Similar to MDS, and despite the absence of evidence from prospective and retrospective trials, pre-transplant treatment with hypomethylating agents or intensive chemotherapy is generally recommended for CMML-2 and CMML with severe proliferative features and/or extramedullary infiltration not controlled by conventional cytoreductive drugs, whereas upfront transplantation may be the preferred strategy for patients with low blast percentages. Recently, a ‘CMML transplant score’, including ASXL1 and/or NRAS-mutated genotype together with BM blasts >2% and co-morbidities, has been proposed to facilitate personalised counselling for CMML candidates for allo-HCT [89].

#### Chronic lymphocytic leukaemia (CLL)

The introduction of signalling pathway inhibitors (PIs), such as the Bruton’s TKI, ibrutinib, the phosphatidylinositol-3-kinase inhibitor, idelalisib, or the BCL2-inhibitor venetoclax, has changed management algorithms and HCT indications in CLL. EBMT and ERIC (European Research Initiative in CLL) have proposed a revised definition of high-risk CLL driven by TP53 abnormalities and response to treatment with PIs [90]. Patients with chemoimmunotherapy-resistant CLL but fully responsive to PI (high-risk I) should be treated with these drugs, and allo-HCT remains an option only in selected patients with a low procedure-related risk. Patients with CLL resistant to both chemoimmunotherapy and PI (high-risk II) have exhausted their main pharmacological therapeutic options and should be considered for cellular therapies, including CAR-T cells and allo-HCT, if eligible. Cellular and molecular therapies are not mutually exclusive and could be used synergistically to exploit their full potential.

Patients with CLL and a concomitant MDS and those with clonally related aggressive transformation of CLL should be considered for allo-HCT regardless of treatment stage of their CLL [91].

Auto-HCT is generally not recommended in CLL. However, it could be considered a clinical option in patients with a histological transformation clonally unrelated to CLL [92].

#### Lymphomas

Since December 2015, lymphoma patients are reported to EBMT via the mandatory MED-A as being in ‘true’ CR1 (first CR directly by standard first-line treatment), or as being in ‘first’ CR (achieved by one or more salvage attempts after primary induction failure), clearly segregating patients with different prognoses. Therefore, the recommendations for lymphoma (in Table 2) refer to ‘true’ CR1 if CR1 is mentioned, and CR1 after prior refractoriness is included as CR >1, and, in some lymphoma types transplant indications in CR1 are more restrictive than in previous editions of these recommendations. In most types of lymphoma, retrospective analyses now show comparable results for MSD, MUD and haploidentical donor transplantation with PTCy (as CB and other alternative donors are used quite rarely in this setting, and are not covered by these statements). Therefore, we have now consistency across current lymphoma classifications.

##### Large B-cell lymphoma (LBCL)

LBCL is defined as any entity behaving clinically similar to diffuse large B-cell lymphoma (DLBCL) regarding treatment options within the scope of this guideline, and includes peripheral mediastinal B-cell lymphoma, high-grade B-cell lymphoma NOS (not otherwise specified) and double-hit and triple-hit lymphomas.

The anti-CD19 CAR-Ts, axicabtagene ciloleucel and lisocaptagene maraleucel, show significant improvement in PFS and a strong trend in OS (significant in univariate analysis) in two Phase III clinical studies in high-risk r/r LBCL compared with salvage therapy (ST) followed by auto-HCT [93, 94]. High-risk relapse of DLBCL was defined as remission duration of <12 months after first-line therapy. By the special characteristics of the study design, chemosensitivity of the patients in the experimental arms is unknown. This urges us to introduce a new category ‘untested relapse’ and solely for this situation the results of CAR-T therapy are superior to those of ST followed by auto-HCT. For patients with high-risk relapsed/refractory LBCL, and unknown chemosensitivity, anti-CD19 CAR-Ts, axicabtagene ciloleucel or lisocaptagene maraleucel, will replace auto-HCT as standard of care.

In late chemosensitive relapse of LBCL after first-line therapy including rituximab, auto-HCT remains standard of care, although CAR-Ts also have to be considered for these patients. As real-world data with anti-CD19 CAR-Ts in third-line use [95] have confirmed the results of the pivotal studies [96–98], CAR-Ts are currently the standard of care in this situation.

For patients failing second-line salvage therapies, relapsing after auto-HCT or with refractory disease, allo-HCT remains a clinical option [99–103] after failure of CAR-T, although there are no robust data available yet. The curative potential of auto-HCT for double-hit lymphomas in primary treatment or thereafter is equivocal [104] and cannot be generally recommended. The recommendation for auto-HCT as consolidation after rituximab-containing first-line therapy in high-risk situations (e.g., slow responding patients to first-line therapy defined by interim PET [105]) needs further confirmation by comparative studies but has a role as a valuable clinical option.

The recommendations for primary central nervous system (CNS) lymphoma are maintained. In this DLBCL subset, there is evidence from a number of non-comparative trials and two randomised controlled trials (RCT) that consolidating auto-HCT in first remission is safe and effective, justifying categorisation as S/II [106–109]. The same holds true for synchronous nodal and CNS lymphoma in primary treatment or later [110]. In contrast, there is virtually no data on the efficacy of allo-HCT in this setting, and therefore, it is generally not recommended.

##### Follicular lymphoma (FL)

In the era of therapeutic antibody maintenance, evidence for benefit of HCT in CR1 is lacking in patients with untransformed FL and in those with high-grade transformation who have not received systemic treatment for the underlying FL before the histological transformation. In contrast, consolidation with auto-HCT may be a clinical option in patients with the chemosensitive high-grade transformation of FL, if they had received prior systemic treatment, especially immunochemotherapy. Beyond the potential efficacy of CAR-T in FL, novel drugs, such as idelalisib, have not changed the natural history of the disease, and transplant indications for FL beyond CR1 remain unchanged compared to previous editions [111, 112].

The anti-CD19 CAR-T axicabtagene ciloleucel results in a high number of durable responses in the third and higher treatment line in a non-comparative clinical trial [113]. It is therefore a clinical option in this situation. Further comparative studies are warranted to define the definite role of the treatment of FL.

##### Waldenström’s macroglobulinemia (lymphoplasmacytic lymphoma with IgM gammopathy; WM)

With the advent of more effective novel agents for WM, such as rituximab, purine analogues, proteasome inhibitors, and kinase inhibitors, strategies using first-line auto-HCT are increasingly questionable and are not recommended outside clinical trials [114]. Auto-HCT should be considered as a clinical option in the first relapse and for patients requiring more than one line of therapy to achieve response [115, 116]. Allo-HCT has been advocated as a clinical option for younger individuals with WM with an aggressive clinical course or high-risk disease according to the IPSS [114, 117]. Although a clear definition of aggressive WM is not formally agreed, allo-HCT might be considered in patients with short-lived responses or refractory to immunochemotherapy, proteasome-based treatment and/or kinase inhibitors.

##### Mantle cell lymphoma (MCL)

Ibrutinib has been approved as an effective salvage treatment for patients with relapsed or refractory MCL. However, in the RCT setting progression-free survival of relapsed MCL is modest with ibrutinib [118]. Moreover, prognosis after ibrutinib failure appears to be extremely poor [119]. Therefore, in contrast to CLL, in MCL the advent of targeted drugs, such as ibrutinib, is yet to significantly affect the natural course of the disease and, thus, indications for HCT. However, ibrutinib might be beneficial for bridging patients with MCL to allo-HCT [120]. Studies testing ibrutinib as part of first-line therapy are ongoing. Available evidence does not suggest benefit of allo-HCT in MCL in CR1 [121]. Therefore, upfront allo-HCT in MCL outside of clinical trials is not recommended.

In a non-comparative study, the anti-CD19 CAR-T, brexucabtagene autoleucel, showed a high number of durable responses in patients failing at least two prior therapies including ibrutinib and mostly pretreated with auto-HCT [122]. The difference in terms of PFS and OS compared to other treatment modalities used in this situation as allo-HCT and the relatively low toxicity defines the medicinal product as standard of care in this situation.

##### T cell lymphomas

Peripheral T-cell lymphomas usually carry a very poor prognosis. Allo-HCT is effective in patients with relapsed and refractory disease and recommended as standard of care in patients with chemosensitive relapse, as the only curative modality in this condition. In CR1, however, a prospective randomised trial testing superiority of allo-HCT over auto-HCT was prematurely terminated due to the low likelihood of meeting its primary endpoint [123]. Thus, both auto-HCT and allo-HCT are clinical options as consolidation of first-response, but the ongoing evaluation is warranted.

Primary cutaneous T-cell lymphomas in early stage have an excellent outcome, and HCT is generally not recommended. However, patients with EORTC/ISCL advanced stages IIB to IV have a dismal prognosis with conventional therapy [124–126]. Allo-HCT offers these patients a clinically relevant and persistent graft-versus-lymphoma effect [127–129], which despite the lack of well-designed comparative trials, would suggest this to be an advantageous clinical option for these patients compared to their outcomes with only conventional therapy.

##### Hodgkin lymphoma (HL)

Targeted agents such as brentuximab vedotin and checkpoint inhibitors may shift the transplant algorithms for HL in the future. For now, as in previous recommendations, auto-HCT remains standard of care for patients with relapsed HL chemosensitive to ST, and allo-HCT in those after a failed prior autograft [130–134]. CAR-Ts have been tested in the setting of prospective clinical trials; the few available results do not show convincingly better results than other treatment modalities [135]. Nodular lymphocyte predominant HL comprises only 5% of all patients being diagnosed of HL; HCT is restricted to those high-risk patients with relapsed disease [136].

#### Multiple myeloma (MM)

The development of new agents, such as proteasome inhibitors, immunomodulatory drugs and mAbs, have been major advances and may eventually change the position of HCT in the treatment strategy. Currently, first-line auto-HCT is still the standard of care for newly diagnosed MM patients [137–139]. Although best results are seen in patients achieving good responses prior to HCT, some non-responding patients also benefit from this approach. Age should be considered in conjunction with general health and fitness. TBI should not be used in the conditioning regimen due to increased toxicity without appreciable benefit, and the addition of bortezomib or lenalidomide to conditioning regimens is yet to be proven to improve patient’s outcome [140]. Double or ‘tandem’ autograft has been shown to be superior to one single auto-HCT, although the benefit of the ‘second’ transplant procedure (of the tandem) appears to be restricted to patients with poor risk features [141] not achieving CR or VGPR with the first transplant procedure. Immunomodulatory drugs and bortezomib as post-HCT consolidation and maintenance therapies may be an alternative option for these patients [142–145].

As the vast majority of patients still relapse after auto-HCT, the use of a further, or ‘salvage’ auto-HCT after re-induction therapy is an option and may be of particular benefit in patients achieving a long treatment-free interval of at least 18–24 months after the first transplant [146]. Relapse within 12 months of first auto-HCT is considered a poor risk and is not in favour of alkylator sensitivity [147].

Allo-HCT is a treatment with curative potential but associated with considerable TRM and might be used in selected high-risk patients [148]. The combination of auto-HCT followed by RIC allograft (‘auto-allo’) has shown a survival benefit for high-risk patients, albeit inconsistently in various clinical trials [149–152]. Recently, allo-HCT with PTCy has been shown to be feasible in MM, but relapse is still a problem [153]. Similar to the auto-HCT setting, novel agents may be complementary, and non-redundant therapies should be combined in the management of suitable allo-HCT candidates. Among patients with end-stage renal impairment, sequential allo-HCT has been reported [154].

CAR T-cell therapy has shown promising results in patients with refractory/relapsed MM [155–157]. Idecabtagene vicleucel (ABECMA^TM^) is the first cell-based gene therapy approved by the FDA for adults with relapsed/refractory MM after four or more lines of therapy, including an immunomodulatory agent, a proteasome inhibitor, and an anti-CD38 mAb. In addition, EMA has recommended granting conditional marketing authorisation in the European Union for ABECMA^TM^ for the treatment of adults with relapsed/refractory MM who have received at least three previous therapies, including an immunomodulatory agent, a proteasome inhibitor and an anti-CD38 mAb. Whether CAR T-cell therapy could replace auto-HCT is under investigation in randomised phase III studies (Karmma-3, NCT03651128, and Cartitude -4; NCT04181827).

#### AL amyloidosis

Patients with systemic immunoglobulin-light-chain (AL) amyloidosis without severe heart failure may benefit from auto-HCT [158]. However, the benefit was not confirmed in a prospective randomised trial that included patients with advanced cardiac amyloidosis [159]. Many recently published studies have reported improved early mortality after an appropriate risk assessment and consistently good haematologic responses and impressive long term survival [160, 161]. Cytogenetic aberrations, as *t*(11;14), can also guide therapy [162]. Allo-HCT might be considered as a clinical option in younger patients who relapsed or not responded after auto-HCT and received at least one new drug (lenalidomide or bortezomib) [163].

#### Acquired severe aplastic anaemia (SAA)

HLA-identical sibling allo-HCT is considered the standard of care for adult patients with SAA, although the outcomes decline over the age of 40 [164–166]. In addition to age, careful assessment of co-morbidities prior to HCT should be made to determine fitness for upfront HCT in the age group of 35–50 years. To reduce the risk of chronic GVHD, all patients should receive in vivo T-cell depletion with ATG or alemtuzumab, and BM is the recommended source of stem cells [167–169]. The choice of conditioning regimen also depends on age; patients <30 years old should receive high-dose cyclophosphamide (200 mg/kg), and those aged 30–40 years old, a fludarabine-based regimen with lower dose cyclophosphamide (120 mg/kg). There is no indication for using radiation in the conditioning for MSD HCT.

MUD HCT can be considered as a first-line choice in young patients aged <18 years based on the excellent outcome compared to historical matched controls [170], provided transplant is feasible within the first two months after diagnosis. Alemtuzumab-based conditioning is also recommended in this situation [171]. If the interval to find a suitable MUD and proceed to HCT is predicted to be longer, then immunosuppressive therapy (IST) with ATG (preferably with horse ATG) and ciclosporin with eltrombopag is recommended in adults.

MUD HCT in young and adult patients is indicated after failure to respond to one course of IST, normally assessed at 3–6 months. Age of recipient is also an issue for MUD HCT, and along with assessment of co-morbidities and other patient and transplant characteristics (e.g., CMV status, source of cells, use of ATG, interval from diagnosis to transplant, HLA matching degree), should help in evaluating patients who would benefit most from the procedure. Classically, patients aged up to 30 years within the first year from diagnosis and refractory to immunosuppressive treatment are the best candidates for MUD HCT. Otherwise, a non-transplant approach is recommended (e.g., eltrombopag if not given first line, androgens, second course of immunosuppressive therapy) [166, 168–174]. As in MSD HCT, BM is the recommended stem cell source for MUD HCT in SAA for ATG-based conditioning regimens. Studies are ongoing to determine whether there is any preferred stem cell source for alemtuzumab-based conditioning, which provides excellent results with durable engraftment and low incidence of chronic GVHD in older (>40 years) recipients of allo-HCT for SAA from MSD or MUD.

Alternative donors for allo-HCT (e.g., CB, haploidentical or MMUD) may be considered after failure to respond to immunosuppressive therapy. Excellent results have been reported especially in young patients up to 20 years of age in the absence of MSD or MUD [175–181]. Those studies also included older patients with promising results [182, 183]. Recent data on haploidentical transplantation is promising [182, 183]. The SAAWP has approved protocols for CB and haploidentical HCT in this setting, where seeking advice from a SAA specialist centre is recommended.

Constitutional SAA and BM failure syndromes, including Fanconi anaemia (FA), dyskeratosis congenita (DKC) and other telomere diseases, may not only present in childhood but also in adults, often with more subtle clinical features. All cases of SAA being considered for HCT should have appropriate diagnostic workup, including molecular genetics, to establish or exclude constitutional BMF syndromes, with consideration of any impact on family donors and conditioning regimen (see relevant sections below).

#### Paroxysmal nocturnal hemoglobinuria (PNH)

The introduction of anti-complement therapy with eculizumab changed the natural history of the disease, and allo-HCT became generally not recommended for patients with haemolytic PNH for whom eculizumab is available. Potential indications remain dependent on the individual clinical manifestations: (i) AA/PNH syndrome, that is, PNH occurring in the presence of severe BM failure with a hypocellular BM (using the same criteria for SAA above for age, disease severity, timing of transplant, conditioning regimen and failure to respond to one course of immunosuppressive therapy in case of MUD HCT) and (ii) clonal evolution of PNH to MDS/AM [184, 185]. Patients with poor response to eculizumab who remain severely transfusion dependent may be also considered for HCT, depending on the availability of new proximal complement inhibitors. Expert advice should be sought from a PNH specialist centre.

#### Solid tumours

Currently, the EBMT Registry includes >63,000 HCT procedures in >46,000 patients with solid tumours, with >7000 procedures performed in the last 5 years. However, with the possible exception of patients with germ cell tumours (GCT), and highly-selected patients with breast cancer (BC), sarcoma and medulloblastoma, HCT is generally not recommended or developmental for most indications in solid tumours [111]. With very limited evidence published recently, the new recommendations in 2022 have changed little compared to prior indications.

Despite the encouraging role of immune surveillance and immune responses against several solid tumours [186–189] recommendations for allo-HCT, as for other forms of cellular therapy, still require further prospective trials, which should be a priority for medical oncology [190, 191].

The role of auto-HCT in BC at high-risk of recurrence and metastatic has been assessed by several randomised trials and meta-analyses of individual patient data [192, 193]. As discussed in more detail in previous reports [111, 194], the overall conclusion is that auto-HCT in BC improves PFS but not OS in most studies. However, auto-HCT may still represent a clinical option for selected patients with specific biological characteristics and/or having gross involvement of axillary nodes (adjuvant setting) or highly chemosensitive disease (advanced setting) [195–199].

In GCT, auto-HCT is a standard of care for patients with disease refractory to platinum-based chemotherapy or with a second or further relapse, a clinical option as a second line in high-risk patients, and generally not recommended as first-line therapy [200–202]. Finally, auto-HCT can be regarded as a potential clinical option in selected patients with Ewing’s and soft tissue sarcomas, medulloblastoma [203–205].

Auto-HCT, being per se capable of inducing marked and rapid tumour regression, may still represent a treatment modality for selected chemosensitive solid tumours and worthy of further study in combination with effective target agents, including immunotherapy. While awaiting results of further prospective trials, the EBMT registry remains an important source to survey indications, outcome and clinical risk factors in patients with solid tumours treated with auto- and allo-HCT.

#### Autoimmune diseases (AD)

Autologous and allo-HCT represent a viable therapeutic approach for many severe AD, resistant to standard therapies, after careful balance of benefits and risks [206]. Most transplant procedures for AD have been performed for multiple sclerosis (MS), followed by systemic sclerosis (SSc), currently representing the two standard indications for auto-HCT in this population [207].

Evidence is continually evolving for auto-HCT, predominantly in MS [208–212], and SSc [213–217], for which auto-HCT can be regarded as a standard of care. There is evidence to support treatment of carefully selected patients with Crohn’s disease [218, 219], systemic lupus erythematosus [220, 221], neuromyelitis optica [222, 223], chronic inflammatory demyelinating polyradiculoneuropathy [224], myasthenia gravis [225], stiff person syndrome [226, 227], systemic vasculitis (ANCA positive [228], Takayasu [229], Behçet’s disease [230]) and refractory coeliac disease [231]. Altogether, available data support considering auto-HCT as an efficacious, one-off intensive therapeutic procedure for severe and refractory AD. Indeed, in the last decade better outcomes have been obtained with auto-HCT, owing to a growing centre experience in selecting the most appropriate patients to transplant paralleled by advances in conditioning and supportive care regimens, accreditation, and national socioeconomic factors [232]. Health economic reports have supported cost-effectiveness in some AD [212, 233, 234].

Despite improved survival over time, allo-HCT has remained predominantly used in younger patients [232]. According to recent EBMT registry data, this strategy can potentially provide long-term disease control in refractory AD, warranting further investigations mainly in younger patients [235]. Syngeneic HCT may be considered as an alternative to auto-HCT. Because autoimmunity can be the main, if not the only manifestation of many monogenic inborn errors of immunity, it is of primary importance to exclude such conditions before considering autologous transplantation, especially in younger adult patients with less well-defined ADs.

Multidisciplinary guidelines and recommendations for both autologous and allo-HCT, across a range of AD, have been published by the EBMT and other professional societies to support clinicians, scientists, as well as patients and carers [232, 236–243] Recently, the EBMT has provided also updated recommendations for the best practice of HCT in AD during the COVID-19 pandemic [236].

#### Inherited diseases in adults

While HCT in inherited diseases is predominantly performed in childhood, adult patients with inherited diseases, including haemoglobinopathies, constitutional BMF syndromes and inborn errors of metabolism (IEM) and immunity (IEI) are increasingly considered for HCT [244]. The indications are the same as for inherited paediatric diseases (covered below), although presentation in adults may differ, including age of onset, course and prognosis. In some cases, access to HCT during childhood has been limited by health service resource and other non-clinical factors, necessitating consideration of HCT in adulthood.

### Transplant indications in children and adolescents

Allo-HCT in children and adolescents represents over 20% of overall allo-HCT activity, with a particular use in congenital and non-malignant diseases, many of which are rare. Transplant complications in paediatric patients impact on with the vulnerabilities of the developing child, including development-related organ dysfunction including infertility, delayed hormonal development, growth retardation and a high risk for malignancies in congenital disorders with chromosomal breakage syndromes. Improvements in high-resolution HLA matching for UD, conditioning regimens and supportive care for infectious and non-infectious complications have progressively reduced mortality and encouraged the positioning of allo-HCT particularly in non-malignant indications at an earlier stage in the course of the disease with patients in a better performance status rather than as a ‘last chance for cure’. GVHD remains the major limitation for patients without optimal matched donors. New allo-HCT strategies should improve outcomes for MMAD. The updated 2022 classification of HCT procedures in children and adolescents is shown in Table 3.

**Table 3:** Proposed classification of transplant indications for children and adolescents—2022.

Disease	Disease status and subtypes	MSD allo	MUD allo	MMAD allo	Auto
*Haematological malignancies*					
AML	CR1 (low risk)^a^	GNR/II	GNR/II	GNR/III	GNR/II
	CR1 (high and very high risk)^a^	S/II	S/II	CO/II	GNR/II
	CR2	S/II	S/II	S/II	GNR/II
	>CR2	S/II	CO/II	CO/II	GNR/II
ALL	CR1 (low risk)^a^	GNR/II	GNR/II	GNR/III	GNR/II
	CR1 (high risk)^a^	S/II	S/II	CO/II	GNR/II
	CR2	S/II	S/II	CO/II	GNR/II
	>CR2	S/II	S/II	CO/II	GNR/II
CML	1st CP, failing 2nd or 3rd line TKI	S/II	S/II	CO/II	GNR/III
	Accelerated phase, blast crisis or >1st CP	S/II	S/II	CO/II	GNR/III
MDS and JMML		S/II	S/II	CO/III	GNR/III
NHL	CR1 (low risk)	GNR/II	GNR/II	GNR/II	GNR/II
	CR1 (high risk)	CO/II	CO/II	CO/II	CO/II
	CR2	S/II	S/II	CO/II	CO/II
HL	CR1	GNR/II	GNR/II	GNR/II	GNR/II
	1st relapse, CR2	CO/II	CO/III	CO/III	S/II
*Non-malignant disorders and solid tumours*					
Primary ID	SCID	S/II	S/II	S/II	NA
	Non-SCID CID	S/II	S/II	S or CO/II	NA
	Primary HLH	S/II	S/II	S/II	NA
	Other primary ID	S/II	S/II	CO/II	NA
MPS	MPS-1H	S/II	S/II	S/II	NA
	Wolman disease^b^	CO/III	CO/III	CO/III	NA
	MPSII–VII^b^	CO/II	CO/II	CO/II	NA
	MLD	S/II	S/II	CO/II	
PSD	X-ALD	S/II	S/II	CO/II	NA
Thalassaemia and SCD		S/II	CO/II	CO/II	NA
Osteopetrosis		S/II	S/II	S/II	NA
IBMFS		S/II	S/II	CO/II	NA
Acquired SAA		S/II	S/II	CO/II	NA
Germ cell tumours		CO/II	CO/II	CO/II	CO/II
Sarcoma	Ewing’s sarcoma (high risk or >CR1)	D/II	D/III	D/III	S/II
	Soft tissue sarcoma (high risk or >CR1)	D/II	D/II	D/III	CO/II
	Osteogenic sarcoma	GNR/III	GNR/III	GNR/III	D/II
Neuroblastoma	High risk or >CR1	CO/II	CO/II	D/III	S/II
Brain tumours		GNR/III	GNR/III	GNR/III	CO/II
Wilms’ tumour	>CR1	GNR/III	GNR/III	GNR/III	CO/II
AD	Including monogenic AD	CO/II	CO/II	CO/II	CO/II

This classification does not cover patients for whom a syngeneic donor is available.

*AD* autoimmune disorders, *ALL* acute lymphoblastic leukaemia, *Allo* allogeneic transplantation, *AML* acute myeloid leukaemia, *Auto* autologous transplantation, *CML* chronic myelogenous leukaemia, *CO* clinical option (can be carried after careful assessment of risks and benefits), *CR1, 2* first, second complete remission, *D* developmental (further trials are needed), *GNR* generally not recommended, *HL* Hodgkin lymphoma, *HSCT* haematopoietic stem cell transplantation, *IBMFS* inborn marrow failure syndromes (Fanconi anaemia, dyskeratosis congenita, Blackfan–Diamond anaemia and others), *ID* immunodeficiency, *JMML* juvenile myelomonocytic leukaemia, *MDS* myelodysplastic syndromes, *MLD* metachromatic leukodystrophy, *MMAD* mismatched alternative donors (cord blood, haploidentical and mismatched unrelated donors), *MPS* mucopolysaccharidosis, *MSD* matched sibling donor, *MUD* well-matched unrelated donor (8/8, 10/10, or 9/10 if mismatched is in DQB1), *PSD* peroxisomal storage diseases, *S* standard of care (generally indicated in suitable patients), *SAA* severe aplastic anaemia, *SCD* sickle cell disease (high risk), *SCID* severe combined immunodeficiencies, *X-ALD* X-linked adrenoleukodystrophy.

^a^Categories are based on number of white blood cells, cytogenetics and molecular markers at diagnosis and time to achieve remission (see text).

^b^For Wolman disease, MPSII and VII, decision is individualised after expert evaluation.

#### Acute myeloid leukaemia

Childhood AML is a rare and heterogeneous disease, with increasing rates of cure and survival with intensive chemotherapy, particularly for patients with favourable prognostic markers. Thus, allo-HCT is not recommended as front-line therapy in low-risk patients, but remains a standard of care for patients in CR1 with high and very high-risk disease with a well-matched donor [245–248]. Alternative donors, in particular, haploidentical family members, have also an increasingly relevant role in high and very-high risk childhood AML and in patients beyond CR1 [249, 250]. Children who experience relapse of AML and reach a second CR are candidates for allo-HCT from the best available donor. Auto-HCT in this setting is generally not recommended outside prospective trials [251].

#### Acute lymphoblastic leukaemia

Allo-HCT from MSD and MUD is a standard of care for high-risk ALL patients in CR1 and in CR2 or later [252–256]. While classical risk factors have included molecular markers, chromosomal abnormalities and biological factors and resistance to initial chemotherapy [257], MRD has now become the most important prognostic factor to discriminate high- and very-high ALL risk groups [258–260]. If an MSD or an MUD cannot be identified, MMAD such as CB, MMUD, or haploidentical family donors are a clinical option [261]. In contrast to adults, stem cells from PB show no advantage in engraftment or relapse incidence compared to BM and therefore BM is the preferred stem cell source for children [262]. Most recently the FORUM trial (EudraCT: 2012-003032-22; ClinicalTrials.gov: NCT01949129), assessed in a non-inferiority study including 417 patients the role of TBI in ALL. Surprisingly, in the intention-to-treat population, the 2-year OS was significantly higher following TBI versus chemotherapy-only conditioning. Two-year cumulative incidence of relapse and TRM were 0.12 and 0.02 following TBI and 0.33 and 0.09 following chemotherapy-only conditioning, respectively. This trial ‘regrettably’ showed that improved OS and lower relapse risk were observed following TBI plus etoposide compared with chemotherapy-only conditioning. At this point in time, TBI plus etoposide must be recommended for patients >4 years old with high-risk ALL undergoing allo-HCT [263].

#### Chronic myeloid leukaemia

As discussed earlier for adult patients, since the advent of TKI, allo-HCT is not recommended as a first-line treatment of CML in children and adolescents either. However, it remains a standard option for patients with treatment failure, recurrence after receiving salvage second-generation TKI treatment and advanced phase CML [264–266]. Of particular relevance for paediatric patients, the indication for allo-HCT needs careful individual consideration to balance the well-established long-term complications of HCT with adverse events from prolonged TKI treatment, which may include growth failure, hepatic, and cardiac complications [267–269]. Stronger evidence from prospective cooperative studies is needed to address disease evolution after TKI discontinuation and other issues specifically in paediatric patients with CML [265, 270].

#### Myelodysplastic syndromes and juvenile myelomonocytic leukaemia

Allo-HCT from a MSD or a MUD is the treatment of choice for children with primary MDS including juvenile myelomonocytic leukaemia, as well as secondary AML [271–273]. Auto-HCT is not recommended outside clinical trials.

#### Lymphoma

Nearly all children and adolescents with Hodgkin and non-Hodgkin lymphoma are cured with multidrug chemotherapy. Few such paediatric patients are eligible for HCT (Table 3) [274–278], but include patients with residual disease after re-induction therapy of contemporary chemotherapy-protocols, patients with early NHL-relapses or patients with inadequate response or relapse of ALK-positive anaplastic large cell lymphoma. All other approaches should be discussed with experts in the front-line chemotherapy trials.

#### Solid tumours

Although the published results have not proven yet an unequivocal benefit for most indications, children and adolescents with solid tumours can undergo auto-HCT following high-dose chemotherapy as a clinical option or within research protocols, preferably as part of first-line treatment strategies (Table 3). Neuroblastoma (stage 4 beyond the age of 1 year, or high-risk factors in lower stages) is still the only indication where the benefit of auto-HCT has been demonstrated by randomised trials [279, 280]. In general, allo-HCT in children with solid tumours should only be explored within prospective clinical trials in highly experienced centres.

#### Acquired severe aplastic anaemia

Allo-HCT from an MSD is the standard front-line therapy for children with acquired SAA. In patients without an MSD, a well-matched unrelated HCT is now also considered a standard front-line therapy in many patients if the donor is readily available, and the search should in any case be initiated before starting any immunosuppressive therapy [281–285]. For those who fail their first course of immunosuppression, if a well-matched UD is identified, the transplant or the second course of immunosuppression should be given, according to clinical status.

#### Autoimmune diseases

Autologous and allo-HCT may be considered a clinical option for children and adolescents with AD [206, 232, 236]. Overall, given the overlap between autoimmune, auto-inflammatory and inborn errors of immunity in the paediatric age group, there should be appropriate specialist expertise in diagnostics (such as NGS, WGS/exome sequencing) and appraising alternative treatment options in the selection of patients for HCT. Special consideration should be given to AD that remains refractory to several lines of conventional and disease-modifying treatments for which an allo-HCT might be appropriate as the ultimate chance for disease control and cure [235].

Auto-HCT may be considered for carefully selected subpopulations of patients with juvenile inflammatory arthritis (e.g., polyarticular course or onset, inadequate response and/or intolerance to prednisone or disease-modifying antirheumatic drugs) and other ADs including systemic sclerosis, systemic lupus erythematosus, vasculitis and polymyositis-dermatomyositis. Paediatric multiple sclerosis is a rare indication of auto-HCT, and long-term responses have been reported [286].

Crohn’s disease is a potential indication for auto-HCT. However, there should be careful consideration of monogenic forms of inflammatory bowel disease (e.g., IL-10 signalling defects, immunodysregulation polyendocrinopathy enteropathy X-linked—IPEX [287]—syndrome, Wiskott–Aldrich syndrome or increasingly X-linked inhibitor of apoptosis—XIAP–deficiency] [240], which are IEI for which allo-HCT is appropriate.

Auto-HCT and allo-HCT have both been performed in severe autoimmune cytopenias, with similar outcomes [288]. Allo-HCT may also result in long-term responses in severe juvenile inflammatory arthritis [289]. A recent retrospective EBMT study reported the long-term outcome of allo-HCT in various haematological and non-haematological severe AD, including also paediatric patients [235]. Better transplant outcomes have been reported for age <18 years, and more recent years of transplant.

#### Inherited diseases

Allo-HCT for inherited diseases features predominantly in the paediatric and TYA group, but occasionally older patients require consideration and treatment. In recent years, genetically modified autologous HCT has become available in some diseases, although detailed appraisal is beyond the scope of these recommendations.

##### Constitutional bone marrow failure syndromes

Allo-HCT is the only treatment able to restore normal haematopoiesis in these patients. Transfusion-dependent FA patients with a suitably well-matched family or UD should be transplanted while in the phase of moderate cytopenia with no poor-risk clonal abnormalities and no MDS/AML [290–292]. For patients who lack a well-matched donor, HCT from MMAD should be considered as a clinical option in the context of a clinical protocol. Although outcomes are reported to be better at age <10 years, this is not the only criterion for decision making. Details on transplant conditioning for particular indications are beyond the scope of these recommendations, but it is important that standard doses of chemotherapy and/or irradiation are absolutely avoided in HCT for FA due to the underlying defect in DNA repair. Although radiation-free regimens including busulfan, cyclophosphamide, fludarabine, ATG with the infusion of a T-cell-depleted graft provide excellent outcomes in HCT from allogeneic donors other than MSD [292], the addition of low-dose irradiation may be indicated for those patients with clonal evolution or receiving transplantation from a UD due to a higher risk of graft rejection. In addition, MSD must be tested for chromosomal fragility, given that some FA subjects can have nearly normal somatic and haematological phenotype. BM is recommended above PB as HSC source, as PB is an independent risk factor for second malignancies.

Patients with DKC and other inherited BMF syndromes should be transplanted if they have a MSD or a MUD [293–295]. A recent large retrospective SAAWP study on allo-HCT for DKC and other telomeropathies showed that pre-transplant organ damage (lung and liver) was associated with poorer outcome [293], supporting thorough organ assessment before HCT. RIC regimens incorporating fludarabine are currently recommended [294, 295]. Potential sibling donors should be tested for telomere length and for mutations of gene of the telomerase-shelterin complex to rule out alterations despite normal somatic and haematologic phenotype.

Patients with Blackfan–Diamond anaemia with a MSD should be transplanted if they do not respond to steroids. If a MSD is not available, allo-HCT may be performed with a MUD in experienced centres [296].

Discussion with a specialist centre is advised regarding possible HCT in patients with constitutional BMF.

##### Inborn errors of immunity (IEI)

IEIs are a large group of >450 genetic diseases characterised by a variable susceptibility to infections and/or immune dysregulation related manifestations such as autoimmunity and autoinflammation [297]. Chronic benign or malignant lymphoproliferation, frequently driven by persistent viral infections can occur and the incidence of malignancies is also increased. Allo-HCT is curative for adaptive immune defects and for selected innate immune deficiencies. Indication and modalities including timing of HCT are highly variable depending on the disease [298]. The decision to proceed with allo-HCT is straightforward in certain conditions such as severe combined immunodeficiencies (SCID) and primary hemophagocytic lymphohistiocytosis (HLH), while for other diseases the final decision can be challenging and depends on a combination of factors, such as immunological parameters, severity of clinical manifestations, organ damage (present or anticipated) and availability of a suitable donor. If the molecular diagnosis is known, insight into the natural history of the disease can help even though a strict genotype/phenotype correlation is often absent. In any case, a careful multidisciplinary evaluation of each individual patient by teams with experience in the care of IEIs is mandatory to tailor the best treatment and guide the patient to HCT with the appropriate timing.

IEIs affecting T-cell immunity with abnormal development and/or function are the most severe and allo-HCT is frequently indicated. SCID is the most severe disease category and leads to an early death unless HCT or in certain circumstances gene therapy, is offered early in life.

Non-SCID-T cell deficiencies are numerous and responsible for a broad variability of symptoms and severity. In the most severe conditions such as MHC class II deficiency [299] Wiskott–Aldrich syndrome, DOCK8 deficiency [300], CD40 ligand deficiency [301], CD27/70 deficiency [302] and a growing number of related diseases allo-HCT has to be strongly considered early in life, particularly if an HLA-matched donor is available. Progress in haploidentical transplants thanks to selective depletion of the graft (such as TCRab/CD19) [303–305] or T-replete graft with PTCy further enlarge indications in selected cases [306, 307].

An increasing number of diseases known as primary immune regulation disorders have been identified. These conditions are treatable with allo-HCT but decision and timing of the transplant are challenging. Control of inflammatory features before transplantation is important [308].

Primary HLH, regardless of the underlying genetic cause is an unambiguous indication for HCT, including with alternative donors. Disease remission prior to allo-HCT is a key factor influencing OS. Pre-emptive HCT, before the manifestation of any HLH-related symptoms, can be discussed in selected cases [309, 310]. Among innate immune defects, complete leukocyte adhesion deficiency has a straightforward indication of HCT [311]. In chronic granulomatous disease, improvement of outcome after HCT allows us to enlarge the HCT indication in particular when an HLA-matched donor is available [312].

Stem cell gene therapy has been pioneered in X-linked and ADA-SCID, leading to EMA licensing of gammaretroviral product Strimvelis®, while excellent results have recently been reported with a lentivirus-based approach [313]. The most recent lentiviral gene therapy studies in other IEI have provided encouraging results and positioning of this alternative curative treatment option is expected in the next years [314, 315].

##### Inborn errors of metabolism (IEM)

Allo-HCT is effective in well-selected patients with peroxisomal diseases (PSD), lysosomal storage diseases (LSD), and some other IEM disorders [316]. Among LSD, success of this approach is best exemplified in type I mucopolysaccharidosis (or Hurler’s syndrome), a severe multisystemic disease with progressive neurocognitive involvement. Engraftment of donor-derived myeloid cells, including microglia, provides the missing enzyme to the recipient. Early intervention (before the age of 2 years), with HCT from a wild-type donor (heterozygous carriers will provide less enzyme), with tailored busulfan-based conditioning regimen to reach full donor chimerism gives the best outcome. CB and BM are both eligible as HSC source. HCT will attenuate but not erase long-term disease manifestations.

In metachromatic leukodystrophy (MLD), a central and peripheral demyelinating disease, allo-HCT is ineffective in early infantile disease but may have an impact in attenuating juvenile and adult forms when applied early in the disease course. Allo-HCT also has a role in other LSD, such as MPSII, MPSVII and Wolman disease. Given the rarity, expert MDT assessment of individual cases is essential.

Among PSD, allo-HCT is effective at preventing disease progression in the childhood, or rarely adult, presentation of cerebral inflammatory disease of X-linked adrenoleukodystrophy (X-ALD). Not all males within kindreds are affected, and allo-HCT should not be offered to those with advanced disease, as it further likely has no impact on other disease manifestations including the later development of myelopathy. Typically, genetically affected males are screened with serial MR imaging and transplant is offered at the earliest sign of disease.

Mitochondrial neurogastrointestinal encephalomyopathy is a rare multisystem disorder caused by mutations in thymidine phosphorylase [317]. Allo-HCT provides the deficient enzyme and prevents further accumulation of toxic substrate responsible for disease manifestations, and progression of the disease. Allo-HCT is often challenging, but considered ‘standard’ in correctly selected clinical cases.

Gene therapy from gene-corrected autologous HSC is very promising in IEM through providing supra-physiologic enzyme production. This is now established therapy in late infantile MLD and trials are in progress in MPSIH and MPSIIIA [318]. Lentiviral GT product have also been recently licensed by EMA in X-ALD [319]. However, careful clinical case selection is needed.

##### Haemoglobinopathies

Allo-HCT from a healthy MSD or a related CB represents the treatment of choice for young patients with transfusion-dependent ß-thalassaemia (TDT). For patients without an MSD, a transplant from a MUD is a clinical option [320–324].

Li et al. [325] showed in TDT that MSD and MUD produce overlapping OS and event-free survival curves so that these donor choices are equivalent, similar to malignant diseases. Although the outcome of haploidentical HCT was inferior in the Li trial, it was probably biased as age is a significant denominator and the value of haploidentical HCT needs to be tested in a controlled stratified trial. HCT in TDT from haploidentical related donors is now increasingly performed as a clinical option in experienced centres [326–328].

The situation in sickle cell disease (SCD) is slightly different to TDT. The mortality of infants and small children with SCD has reduced significantly with simple measures of conventional therapy such as vaccination, antibiotic treatment, parent education and the use of hydroxyurea starting in infancy. Nevertheless, adult mortality has only shifted to older ages as conventional therapies do not have an impact on progressive organ damage. Therefore, adults continue to succumb to heart, pulmonary, renal failure and stroke, and remain often disabled many years prior to these terminal events. For this reason, allo-HCT from a MSD is standard of care and should be offered prior to the emergence of serious complications [329–331].

In SCD, age is a significant denominator for outcome since the incidence of acute and, more significantly, chronic GVHD reaches 17 and 20%, respectively, following HCT from a MSD in patients beyond 15 years [332]. The general theme that MSD and MUD produce overlapping OS and event-free survival is not reproducible in SCD where HCT from MUD is inferior to MSD with a major decline in outcome in patients beyond 13 years [333].

In SCD, MSD/MUD donor availability is below 20%. Recent reports demonstrated successful outcomes with haploidentical transplantation [334, 335] and alternative donors are considered a clinical option using either PTCy or T-cell-depleted strategies.

Since SCD is mainly a systemic vasculopathy with unexpected and disease-specific complications during allo-HCT such as PRES, macrophage activation syndrome, significant pain crises during HCT and organ failure, experimental approaches should only be performed in controlled clinical trials in highly experienced centres [334].

CRISPR-Cas9 gene-editing approaches are currently evaluated in Phase I trials as potential alternatives to allo-HCT [336]. Lentiviral-based gene therapy approach for beta-thalassaemia and SCD have received market authorisation but are not available in Europe.

Importantly, HCT for hemoglobinopathies should be performed early in life to reduce complications, in particular the incidence of chronic GVHD. Delay may also lead to irreversible damage due to iron overload in patients with TDT and systemic vasculopathy in patients with SCD.

##### Osteopetrosis (OP)

OP is a heterogeneous genetic disease related to several gene defects affecting osteoclast function and characterised by impaired bone resorption. Bone density is increased on X-ray. Classical infantile OP leads to early manifestations such as macrocrania, vision impairment, haematological insufficiency, hepatosplenomagaly and hypocalcemia. Urgent HCT is indicated after exclusion of neurodegenerative and osteoclast-extrinsic defects. Atypical, delayed disease may occur, for whom HCT may be considered in case of haematological insufficiency or imminent visual impairment [337].

## Quality in HCT: JACIE and EBMT benchmarking

Currently, the JACIE standards, produced in collaboration with the Foundation for the Accreditation of Cellular Therapy (FACT), are in their 8th edition and have been expanded to incorporate immune effector and other cell and gene therapies, such as CAR-T cells. JACIE has been increasingly recognised by governmental bodies and competent authorities in several EU member states and extends beyond Europe with accredited centres worldwide. Importantly, JACIE accreditation appears to have an impact on survival outcomes and donor safety [232, 338–344]. During the pandemic, JACIE supported self-assessment by centres to help maintain minimum quality standards in the midst of the challenges faced by health services. How JACIE adapts to the post-pandemic situation is an area for active consideration with greater use of remote inspections, self-assessments and other practicable ways of working.

In terms of ongoing developments, JACIE and the EBMT Registry have delivered the first two pilot phases of a risk-adapted benchmarking system that takes into consideration the heterogeneity of the disease, patient and transplant characteristics. This will enable EBMT member centres to benchmark their survival outcomes against national and/or international norms, irrespective of the size of their HCT community, and potentially this could apply to individual indications to provide ‘real-world’ estimates and benchmarking of survival at international, national or centre level and comparison with non-transplant alternatives [345].

## Conclusions

For over two decades, the EBMT indications reports have incorporated developments in HCT practice based on scientific and technical developments in HCT. We encourage harmonisation of practice, where possible, to ensure meaningfully aggregated experience across indications via registry outputs. We also recommend working according to JACIE accreditation standards benchmarking of outcomes to maintain quality in HCT practice.

Moving forward, all treatment decisions, whether HCT or non-HCT, need to accommodate the ongoing COVID-19 pandemic. Although the full impact of the pandemic is yet to be determined, our understanding and evidence-base is evolving in combination with public health measures, vaccination strategies and new treatments in the HCT population. We therefore recommend that decision making across indications is delivered within the MDT with reference to EBMT and other national guidance in relation to COVID-19, and in accordance with current local conditions.
